# Evaluating the durability of zirconia - based crowns among patients with bruxism

**DOI:** 10.6026/973206300212500

**Published:** 2025-08-31

**Authors:** Shaivi Sharma, Pradyumna Kumar Sahoo, Arun Kharavela Mohanty, Pooja Soni, Ashtha Arya, Bhavana Soni

**Affiliations:** 1 Department of Dentistry, Amaltas Institute of Medical Science, Bangar Village, Ujjain Dewas road Dewas Madhya Pradesh, India; 2 Department of Prosthodontics and Implantology, Institute of Dental Sciences Siksha O Anusandhan University, Bhubaneswar Odisha, India; 3 Department of Prosthodontic Crown and Bridge, Kalinga Institute of Dental Sciences, Bhubaneswar, India; 4 Department of Prosthodontics, College of Dental Science and Hospital, Rau, Indore, Madhya Pradesh, India; 5 Department of Conservative Dentistry and Endodontics, SGT Dental College, Hospital and Research Institute, SGT University, Gurugram, Haryana, India; 6 Department of Oral and Maxillofacial Pathology & Microbiology, Surendra Dental College and Research Institute, Sriganganagar, Rajasthan, India

**Keywords:** Zirconia crowns, bruxism, dental restorations, crown durability, occlusal forces, veneer chipping, marginal adaptation

## Abstract

The clinical durability of zirconia-based crowns in patients with bruxism over a 12-24 month period is of interest. Patients received zirconia crowns and were assessed for crown fracture, veneering chipping, marginal adaptation, wear and satisfaction. The crowns showed high structural integrity with minimal complications. Minor chipping of veneering porcelain was observed, but no fractures or debonding occurred. Thus, zirconia-based crowns maintained function and esthetics under high occlusal stress.

## Background:

Bruxism is a Para functional habit characterized by involuntary grinding or clenching of teeth, often occurring during sleep, which can lead to significant wear and damage of dental structures and restorations. It affects both natural dentition and prosthetic work, posing a challenge for long-term restorative success [1]. The increased occlusal forces generated in bruxism can lead to failure modes such as crown fracture, veneering porcelain chipping and marginal breakdown. Zirconia-based crowns have emerged as a widely used material in prosthodontics due to their favorable mechanical properties, including high fracture toughness, flexural strength and biocompatibility [2, 3]. Unlike metal-ceramic restorations, zirconia offers a metal-free, esthetically pleasing solution while maintaining durability under stress. However, their performance in patients with bruxism remains a subject of clinical interest, especially given the potential for chipping of veneering ceramics and surface wear under high occlusal loads [4]. Therefore, it is of interest to describe the clinical durability of zirconia-based crowns in patients with bruxism over an extended follow-up period, focusing on structural integrity, esthetic preservation and patient satisfaction.

## Methodology:

This prospective clinical study was conducted on a total of 20 patients diagnosed with sleep or awake bruxism, aged between 25 and 55 years, who required full-coverage single-unit crowns in posterior teeth. Ethical approval was obtained from the institutional review board and written informed consent was acquired from all participants prior to treatment. All selected patients underwent thorough clinical and radiographic evaluation. Bruxism was confirmed through patient history, clinical signs (such as wear facets and masseter hypertrophy) and self-reporting or partner confirmation of nocturnal grinding. Patients with temporomandibular disorders, uncontrolled systemic diseases, or poor oral hygiene were excluded. Each participant received a monolithic zirconia crown (Yttria-stabilized tetragonal zirconia polycrystals - Y-TZP). Tooth preparations followed standard protocols, ensuring 1.5 mm occlusal reduction and 1 mm axial reduction with a chamfer finish line. Impressions were taken using a polyvinyl siloxane material and crowns were fabricated via CAD/CAM technology. Crowns were cemented using self-adhesive resin cement under rubber dam isolation. Clinical follow-up assessments were carried out at 6, 12 and 24 months post-cementation. Evaluations included crown integrity (fracture or chipping), marginal adaptation (using explorer and radiographs), occlusal wear (visual inspection) and patient-reported satisfaction (via a standardized questionnaire). Any biological or technical complications were recorded. All data were collected by the same calibrated clinician to minimize operator variability. The primary outcome was the presence or absence of structural failure. Secondary outcomes included chipping, surface wear, marginal changes and patient satisfaction. Data were statistically analyzed using descriptive methods and chi-square testing, with a significance level set at p < 0.05.

## Results:

A total of 20 patients (12 males, 8 females; mean age 39.5 ± 8.3 years) completed the 24-month follow-up. None of the patients reported crown loss, debonding, or secondary caries during the study period. No complete crown fractures were observed in any of the 20 zirconia crowns placed and minor veneering ceramic chipping occurred in only 2 cases (10%), which were managed with intraoral polishing (Table 1 - see PDF). Chipping was limited to maxillary and mandibular molars, with no incidents in premolars (Table 2 - see PDF). Marginal integrity remained clinically acceptable in 95% of cases at 24 months, with only one case showing a minor discrepancy; no major defects were observed throughout the study duration (Table 3 - see PDF). Gingival response was healthy in 90% of patients and no adverse periodontal changes were noted. Patient-reported outcomes were highly favorable. At 24 months, 90% of participants rated both functional and esthetic outcomes as "very satisfactory." Similarly, 85% reported very satisfactory comfort and 95% expressed a strong willingness to recommend zirconia crowns (Table 4 - see PDF).

## Discussion:

The clinical performance of zirconia-based crowns in patients diagnosed with bruxism over a 24-month period. The results showed that zirconia crowns demonstrated excellent durability under increased occlusal loads, with no complete crown fractures and only minor incidences of veneering ceramic chipping. These findings are consistent with previous research highlighting zirconia's high fracture resistance and mechanical stability in functionally demanding environments [5, 6]. The absence of crown fractures in all 20 cases over a two-year period reflects zirconia's inherent material strength, which has been attributed to its transformation toughening mechanism and high flexural strength exceeding 900 MPa [7]. Beuer *et al.* (2016) highlighted that zirconia-based crowns exhibit superior fracture resistance under cyclic loading, making them suitable for high-stress conditions like bruxism. Their study demonstrated minimal wear and structural degradation over time. This supports the clinical use of monolithic zirconia in bruxism patients due to its high durability [8]. Marginal integrity remained intact in 95% of cases, suggesting that zirconia crowns maintain their adaptation well over time, likely due to minimal marginal degradation and stable cementation interfaces [9]. This is clinically significant, as compromised margins can lead to secondary caries and periodontal inflammation. The periodontal response in this study was favorable, aligning with literature that supports zirconia's biocompatibility and low plaque accumulation compared to metal-ceramics [10, 11]. Patient satisfaction levels were high in terms of function, esthetics and comfort. Previous reports have also confirmed that zirconia's tooth-like color, absence of metal margins and smooth surface texture contribute positively to patient perceptions and acceptance [12]. Additionally, bruxism patients are often concerned about restoration durability; therefore, the positive outcomes reported here are clinically encouraging and consistent with other mid-term studies [13]. Sulaiman *et al.* (2021) found that monolithic zirconia crowns maintained structural integrity and functional performance even under bruxism-induced stress. The study emphasized the material's high flexural strength and low wear potential. These findings reinforce zirconia's suitability for long-term use in bruxism patients [14]. Gubrellay *et al.* (2025) evaluated the clinical longevity of zirconia crowns in bruxism patients and reported promising outcomes over time. The crowns showed minimal fracture or surface degradation despite high occlusal forces. This supports zirconia's resilience and effectiveness in managing bruxism-related stress [15]. Transitioning to fully monolithic zirconia crowns has been proposed as a strategy to minimize veneer-related failures, particularly in bruxers.

## Conclusion:

Zirconia-based crowns demonstrated excellent durability and clinical performance in patients with bruxism over a 24-month period. No crown fractures were observed and only minor chipping occurred in a small number of cases. Marginal integrity and patient satisfaction remained consistently high. Thus, we show the reliability of zirconia crowns under high occlusal stress conditions.
